# Epigenomic signatures reveal mechanistic clues and predictive markers for autism spectrum disorder

**DOI:** 10.1038/s41380-022-01917-9

**Published:** 2023-01-17

**Authors:** Janine M. LaSalle

**Affiliations:** Department of Medical Microbiology and Immunology, Perinatal Origins of Disparities Center, MIND Institute, Genome Center, Environmental Health Sciences Center, University of California Davis, Davis, CA USA

**Keywords:** Autism spectrum disorders, Genetics

## Abstract

Autism spectrum disorder (ASD) comprises a heterogeneous group of neurodevelopmental outcomes in children with a commonality in deficits in social communication and language combined with repetitive behaviors and interests. The etiology of ASD is heterogeneous, as several hundred genes have been implicated as well as multiple in utero environmental exposures. Over the past two decades, epigenetic investigations, including DNA methylation, have emerged as a novel way to capture the complex interface of multivariate ASD etiologies. More recently, epigenome-wide association studies using human brain and surrogate accessible tissues have revealed some convergent genes that are epigenetically altered in ASD, many of which overlap with known genetic risk factors. Unlike transcriptomes, epigenomic signatures defined by DNA methylation from surrogate tissues such as placenta and cord blood can reflect past differences in fetal brain gene transcription, transcription factor binding, and chromatin. For example, the discovery of *NHIP* (neuronal hypoxia inducible, placenta associated) through an epigenome-wide association in placenta, identified a common genetic risk for ASD that was modified by prenatal vitamin use. While epigenomic signatures are distinct between different genetic syndromic causes of ASD, bivalent chromatin and some convergent gene pathways are consistently epigenetically altered in both syndromic and idiopathic ASD, as well as some environmental exposures. Together, these epigenomic signatures hold promising clues towards improved early prediction and prevention of ASD as well genes and gene pathways to target for pharmacological interventions. Future advancements in single cell and multi-omic technologies, machine learning, as well as non-invasive screening of epigenomic signatures during pregnancy or newborn periods are expected to continue to impact the translatability of the recent discoveries in epigenomics to precision public health.

## Introduction

Autism spectrum disorder (ASD) refers to a complex group of neurodevelopmental disorders characterized by deficits in social communication and language and gains in repetitive and restrictive behaviors and interests. The prevalence of ASD has been steadily increasing over the past 20 years, from US child estimates of 0.66% in 2002 [1], 1.13% in 2008 [2], 1.85 in 2016 [3], and 2.27% in 2018 [4]. Changes over this period in the rate of ASD is in part due to increased awareness and changing diagnoses [5–8]. However, even estimates that account for diagnostic changes still leave an apparent increase that cannot likely be explained by genetics alone [9]. Furthermore, ASD heritability estimates have been discordant in different twin studies, depending on the number of subjects, geographical, and demographic differences [10–13]. While there has been much progress in the discovery of new ASD genes by detection of rare de novo mutations from exome sequencing studies, no single ASD gene can account for more than 1% of ASD [14–18]. Approaches to identify common genetic variants for ASD using genome-wide association studies have revealed a shared genetic architecture with other disorders and traits [19]. Together, these finding have demonstrated that ASD etiology is decidedly complex, involving hundreds of genes and interactions with environmental factors.

Epigenetics, literally meaning “on top of genetics” is a field that investigates additional layers of relevant biological information for interpreting phenotypes that do not alter the genetic code. Poised at the interface of genetic and environmental influences, the investigation of epigenetic modifications such as DNA methylation can reveal novel insights that are not apparent in the DNA sequence. See Box 1 for more details of epigenetic definitions and terms. However, the field of epigenetics is inherently integrated with genetics, as genetic variation frequently influences epigenetic variation [20]. Since the most common heritable variability exists outside of protein coding exons, these variants can be more difficult to interpret without the important context of epigenetics. Epigenetic layers of information have been used to functionally annotate the human genome with activity levels of promoters and enhancers as well as chromatin loops and domains of similar DNA methylation levels [21–24]. Since environmental factors act by altering responsive gene expression patterns, these can leave distinctive “signatures” at the level of epigenetic modifications that leave long-lasting effects on gene expression, particularly when the exposures occur in utero or early postnatal life [25, 26].

The field of autism epigenetics began initially through genetics, specifically the investigation of known genetic neurodevelopmental disorders with ASD comorbidity that were epigenetic in their inheritance pattern and/or gene function [27, 28]. These included the disorders of the parentally imprinted gene cluster at 15q11-q13, in which large maternal deletions cause Angelman syndrome but the same deletion inherited paternally cause Prader-Willi syndrome [29–32]. Also of early interest in this field was Rett syndrome, an X-linked dominant disorder affecting females, caused by mutation in *MECP2*, a gene encoding a known epigenetic player, methyl CpG binding protein 2 [33, 34]. Candidate gene approaches to investigate epigenetic differences were also based on expectations from both genetics and neuroscience and include genes such as oxytocin receptor [35]. More recent genome-wide investigations of epigenetic differences in ASD have included epidemiology-based epigenome-wide association studies (EWAS) that most frequently utilize commercial array-based platforms. However, sequencing-based approaches such as whole genome bisulfite sequencing (WGBS) and those using some degree of reduced representation are becoming more common. See Box 2 for an explanation of platform differences in EWAS.

DNA methylation is the most frequently studied epigenetic modification in genome-wide ASD studies for both practical and biological reasons. On a practical level, DNA is much easier to obtain from a variety of limited clinical samples and DNA methylation is a relatively stable mark in archived frozen tissues [36], relative to RNA or histone modifications. But also biologically, DNA methylation in the human genome is highly abundant, correlates with other epigenetic layers including open chromatin and histone modifications, and is the layer closest to and most influenced by DNA sequence [37]. Therefore, this review will focus on investigations of DNA methylation and the signatures of DNA methylation patterns that are emerging and converging between different etiologies of ASD. Starting with a review of the evidence that DNA methylation signatures reflect predominantly gene by environment interactions in general, I will then move to the evidence for individual genetic versus environmental etiologies.

Box 1: What is epigenetic? Definitions and terms **Epigenetic:** Modifications to DNA or chromatin that can alter gene expression and phenotypes without changing the DNA sequence.

**Epigenetic layers:** Epigenetic modifications that exist as specific layers of molecular information on top of DNA, including (in increasing order of distance from DNA): DNA methylation, histone post-translational modifications, chromatin loops, higher-order chromatin compartments, noncoding RNAs acting as chromatin modifiers

**Epigenetic players (or factors)**: Protein factors that modify, recognize, or change epigenetic layers. This group represents over 800 proteins encoded in the mammalian genome [149] and many known ASD risk genes [45, 150]. Specific examples include DNA methyltransferases (*DNMT3A*), demethylases (*TET2*, *TET3*), histone deacetylases (*HDAC4*, *HDAC8*), histone demethylases (*KDM3B*, *KDM5A*), and chromatin remodeling factors (*ATRX*, *SATB1*, *CHD8*).

**DNA methylation base pair targets:** CpG, CpH (H represents A, T, or C)

**DNA methylation modifications:** 5-methylcytosine (5mC), 5-hydroxymethylC (5hmC)

**CpG islands:** clusters of 5’-cytosine-guanine-3’ dinucleotides in mammalian genomes that are frequently unmethylated when occurring at the promoters of active or ubiquitously expressed genes.

**Gene bodies:** regions of genes defined as transcription start site to transcription end site.

**Bivalent chromatin**: developmentally poised chromatin state characterized by both active and repressive histone marks.

**Epigenomic:** Genome-wide analyses of specific epigenetic modifications or chromatin features using microarray (Illumina Infinium 450 K or EPIC) or sequencing (WGBS, chromatin immunoprecipitation sequencing: ChIPseq, Assay for Transposase-Accessible Chromatin: ATACseq, high throughput chromosome conformation capture: HiC) approaches.

**E**pigenome **w**ide **a**ssociation **s**tudy (EWAS): an analysis of cases and controls for differentially methylated regions or probes by array or sequencing-based approaches (See Box 2).

**Variably methylated regions (VMR):** genomic regions defined as having the most interindividual variability of all regions assayed on the array or sequencing platform.

**Differentially methylated probes (DMP):** individual CpG sites detected by probes on the Illumina Infinium arrays (described in Box 2) showing differential methylation between cases and controls.

**Differentially methylated regions (DMR):** genomic clusters of ~3–20 CpG sites with coordinate differential methylation patterns between two comparison groups of samples (ASD cases versus controls) or alleles (maternal versus paternal in imprinted regions).

**Epigenomic signature:** A profile of multiple changes to an epigenetic layer (usually DNA methylation) that together separate ASD from control samples using a principal components or clustering analysis. Like a handwritten signature, each individual part of the signature may not be precisely reproducible every time, but the multiple parts together have a distinctive combination of features that allow bioinformatic clustering by diagnosis. While an epigenomic signature is likely to be secondary to the primary cause(s) of ASD, it can inform both diagnosis as well as dysregulated gene pathways for pharmacological interventions.

Box 2: Genome-wide methylome approaches for detecting epigenomic signatures

- **Array-based methods**
  Commercially available platforms that include hybridization to probes over predefined genomic regions. Sample DNA undergoes bisulfite conversion prior to hybridization to methylated and unmethylated probes for individual CpG sites.
  Illumina Infinium 27k was designed to include probes mostly over known gene promoters.
  Illumina Infinium 450k included some known enhancers as well as promoters.
  Illumina Infinium EPIC expanded to 850k design to include FANTOM5 enhancers, ENCODE open chromatin and enhancers, DNase hypersensitive sites, and miRNA promoter regions.
  - **Advantages**
    ● For human epidemiology studies, having a uniform platform makes it easier for cross-comparisons comparisons and meta-analyses.
    ● Bioinformatic pipelines are well-established and data storage is minimal.
    ● Data integrate with array-based genotyping for determining methylation quantitative trait loci (meQTLs).
    ● Cost is generally less expensive than sequencing-based approaches, but not always (see below).
  - **Disadvantages**
    ● Cover <3% of all CpGs in the human genome.
    ● Only available for human studies.
    ● Can require higher input DNA than current sequencing-based approaches.
    ● Identifying differentially methylated regions (DMRs) is challenging.

- **Sequencing-based methods**
***Whole Genome Bisulfite Sequencing (WGBS)***
  Using the most current library preparation methods, genomic single stranded DNA is fragmented and bisulfite converted prior to ligation of truncated adapters, DNA synthesis, and ligation of indexed bar codes [151]. After alignment to a reference genome, percent methylation is calculated for individual CpGs or clusters of CpGs in differentially methylated regions (DMRs).
  Low pass WGBS varies from 1x-10x coverage genome-wide, which is sufficient for DMR-based analyses, network analyses of comethylated regions [152], as well as global methylation analyses).
  High coverage WGBS varies from 30x-50x coverage genome-wide, which is sufficient for single CpG resolution [153].
  Reduced-representation bisulfite sequencing (RRBS) uses a either a methyl-sensitive restriction enzyme or hybridization-based capture approach to enrich DNA fragments for specific genomic regions prior to bisulfite conversion, library preparation, and sequencing.
  - **Advantages**
    ● WGBS covers >20 million CpGs and RRBS genomic coverage is greater than arrays.
    ● Any species for which a reference genome is available can be analyzed.
    ● WGBS libraries can be generated from 1 ng of DNA.
    ● WGBS CpG coverage is ideal for DMR analyses, as well as comparison to all available epigenomic sequencing maps of transcription factor binding sites and chromatin states.
    ● The cost of genomic sequencing continues to drop, making low-pass WGBS and RRBS approaches comparable to arrays.
  - **Disadvantages**
    ● Comparison of results to published array-based EWAS studies is challenging. For instance, in a recent WGBS cord blood analysis of cord blood, >80% of ASD DMRs did not overlap with even a single probe represented on the Infinium EPIC array [95].
    ● Relatively large data storage and computational needs for bioinformatics
    ● The cost is higher than array-based approaches, but only for high-coverage WGBS.

- ***New developments in bisulfite-free sequencing approaches***
- **Enzymatic methyl-seq** **(EM-seq)** EM-seq utilizes two biological enzymes, first to convert 5mC to 5hmC with TET2, then to deaminate unmethylated cytosine to uracil with APOBEC3A [151, 154]
  - **Advantages**
    ● By eliminating the use of bisufite, DNA is less damaged, allowing DNA inputs down to 100 pg.^40^
  - **Disadvantages**
    ● Enzymatic conversion efficiency is lower than bisulfite, leading to genome complexity problems resulting in low mapping rates and uneven genome coverage^40^

- **Oxford Nanopore sequencing and Nanopolish** directly detects 5 mC from long reads using a Hidden Markov model that generates a log-likelihood value for the ratio of probability of methylated to unmethylated CGs at a specific k-mer. This approach was used to map DNA methylation in the latest telomere to telomere (T2T) human genome [120, 155].
  - **Advantages**
    ● Long reads processed without bisulfite allows repetitive regions of the genome to be investigated.
  - **Disadvantages**
    ● Nanopore sequencing has less accuracy of methylation quantitation at base pair resolution.

### Evidence that DNA methylation predominantly reflects gene by environment interactions in early life

Genetic and environmental factors may act independently on DNA methylation, or in additive or multiplicative interactions. At the simplest level, some genetic variants including single nucleotide polymorphisms (SNPs) can directly determine DNA methylation, for instance, if they cause a gain or loss of a CpG. These are expected to be rare (<1%) based on a study of neonatal genotype-methylation comparisons from cord blood [38]. In this array-based study, only 25% of variably methylated regions were estimated to be explained by genotype alone, and 75% were explained by genotype x in utero environment interactions [38]. A sequencing-based study identified a slightly higher 32% of methylated CpGs as being genetically regulated and enriched in enhancers, compared to ~14% that were not dependent on genotype and enriched in repressed regions and near transcription start sites [39]. A more recent and larger array-based investigation of newborn cord blood determined the best models to explain DNA methylation, concluding that genetic (G), gene plus environment (G+E), and gene by environment interaction (GxE) explained roughly equal proportions of variably methylated regions, and was consistent with previous studies in showing no evidence for environment alone [40]. In this study, variants with best models G, G + E or GxE all showed significant enrichment within GWAS signals for complex disorders beyond the enrichment of the functional variants themselves. ASD strikingly stood out among other neuropsychiatric disorders as having the highest enrichment of GWAS loci enriched in methylation patterns best explained by GxE (Odds ratio > 2) [40].

Variably methylated regions (VMRs) have been investigated in more detail across different human tissues and environmental conditions, revealing some interesting insights. An array-based study examined polymorphic human methylation patterns across five cell types and ages, identifying both unique and common VMRs [41]. Interestingly, these VMRs were found to form co-methylated networks that were enriched for genes and transcription factor binding sites with cell-type relevant functions. In neurons, the top enriched gene function was for “synapse assembly” while in glia it was “negative regulation of neurogenesis” [41], suggesting the influence of VMRs on gene functions relevant to ASD. A sequencing-based approach mapped VMRs across the human genome, estimating that they make up 11% of the genome and are enriched in histone modifications indicative of enhancers, transcription factor binding sites and GWAS variants, including those for ASD [42]. A sequencing-based study identified that the most interindividual differences in DNA methylation between humans occurred in defined correlated regions of systemic interindividual variation (CoRSIVs), defined as having similar DNA methylation patterns across tissue types [43]. While CoRSIVs include genes associated with human disease phenotypes, conserved across diverse human ethnic groups, and sensitive to periconceptional environment, they unfortunately are very sparsely covered on commercial methylation arrays.

Twin studies have been critical to understanding the heritability of DNA methylation patterns in humans. Array-based comparisons of monozygotic (MZ) to dizygotic twins (DZ) using 27k and 450k platforms (Box 2) have estimated heritability of DNA methylation ranging from 3–20% depending on the age of the subjects. A more recent 450k study examined both genetic and environmental contribution of multiple twin cohorts of different ages (0–92 years) by calculating familial correlations of DNA methylation compared to time of twin cohabitation [44]. In this study, familial correlations for 6.6% of CpG sites assayed changed with twin pair cohabitation history and these “cohabitation” sites were enriched for regions with the highest high heritability (31% versus 19% heritability for all sites). These “cohabitation” sites are therefore consistent with the high degree of methylation explained by GxE in prior studies [38, 40], and were similarly enriched for locations of genes involved in nervous system development and genetic associations for cognitive traits [44]. Importantly, the life course design of this twin study provided evidence that early life could affect later life health through DNA methylation. Furthermore, the studies of both twins and unrelated subjects have demonstrated that the CpG sites with the strongest heritability are also those that are also most influenced by environmental factors.

### Evidence for epigenomic signatures of human syndromic neurodevelopmental disorders with ASD

Consistent with distinguishable genetic effects on DNA methylation, syndromic human neurodevelopmental disorders are also emerging as having distinct epigenomic signatures. While these syndromes have known genetic etiologies, many of these involve mutations to modifiers of chromatin [45], so differences in DNA methylation are likely reflecting genome-wide changes in chromatin compared to controls. The discovery of syndrome-specific epigenomic signatures has direct relevance to clinical diagnosis because they offer the possibility of using a single test platform, such as the Illumina Infinium 450k or EPIC array, using blood-derived DNA, to distinguish multiple neurodevelopmental syndromes from each other. But perhaps more importantly, the epigenomic signature in both brain and appropriate surrogate tissues provides specific clues throughout the genome about the downstream effects on genes, transcription factors, and pathways in the molecular pathogenesis of each syndromic disorder.

DNA methylation patterns have been used for decades to diagnose imprinted and X-linked disorders such as Prader-Willi/Angelman syndromes (PWS/AS) and fragile X syndrome because they offered specific tests that would encompass different genetic and epigenetic causes of gene loss or repression. However, a recent study demonstrated that four different imprinted syndromes (PWS, AS, Beckwith-Wiedemann, Silver-Russell) could be distinguished from each other and 364 reference controls with 100% specificity and sensitivity using the 450k array platform from blood DNA [46]. What is new in recent years is the number of genetic syndromes not arising from mutations in epigenetically regulated chromosomal loci that can be predicted based on DNA methylation signatures. In 2019, a clinical DNA methylation assay, “EpiSign,” was introduced for the screening of 14 syndromes using supervised and unsupervised classification algorithms [47]. In 2020, the same group utilized a similar strategy to identify distinct epigenomic signatures in 34 out of 42 neurodevelopmental syndromes [48]. Most of these epigenomically defined syndromes are caused by known ASD candidate genes involved in chromatin regulation (*ADNP*, *ATRX*, *CHD8*, *KDM6A*, *KTM2B*) or DNA methylation (*DNMT1*, *DNMT3A*, *DNMT3B*). Approximately 50 different syndromes caused by genes encoding epigenetic regulators, dubbed “chromatinopathies”, have been clinically defined and the clinical utility of using methylation data and EpiSign for improved diagnosis has been validated by independent teams [49, 50]. While these epigenomic signatures are secondary events in the disease pathogenesis downstream of the causative mutation, they offer insight into disease pathogenesis and well as applications in clinical diagnosis.

**Fig. 1:**
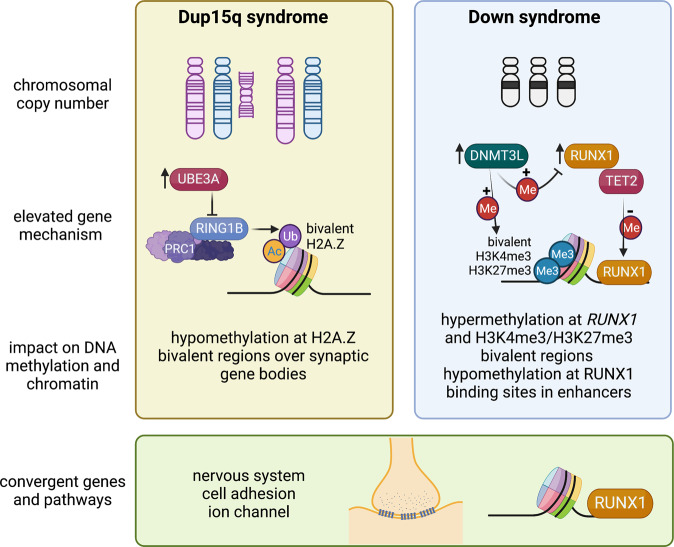
Mechanistic insights from epigenomic signatures of chromosomal syndromes with ASD. Examples are shown of two disorders caused by a large chromosomal duplication, 15q11-q13 duplication syndrome (Dup15q, left panel), or aneuploidy, Down syndrome (trisomy 21, right panel). In Dup15q syndrome, duplications are either supernumerary (left) or interstitial (right) and depend on parent of origin, as only maternal (pink chromosome) duplications are associated with ASD. Elevated levels of the imprinted gene *UBE3A* are predicted to initiate the pathogensis of Dup15q syndrome. UBE3A is an E3 ubiquitin ligase that targets a different E3 ubiquitin ligase called RING1B, part of the PRC1 complex, that monoubiquitinates H2A and H2A.Z, resulting in a maintenance of bivalency (acetylation and ubiquitination). The epigenomic signature of Dup15q brain and neuronal cell line models has revealed hypomethylation at H2A.Z bivalent regions over synaptic genes due to reduced RING1B levels. In contrast, Down syndrome brain is characterized by both hypermethylation and hypomethylation and two chromosome 21-encoded proteins are distinctly implicated in each process. Elevated DNMT3L increases methylation over regions of bivalent chromatin marked by H3K4me3 and H3K27me3, including the chromosome 21 locus *RUNX1*, encoding a developmental transcription factor. The regions hypomethylated in Down syndrome newborn blood were enriched for RUNX1 binding sites, suggesting that RUNX1 targets these sites for demethylation from its known association with TET2. While the mechanisms behind the epigenomic signatures of Dup15q and Down syndromes are distinct, synaptic gene pathways are apparent in both and the *RUNX1* locus shows differential methylation in both syndromes. Created with BioRender.com.

In addition to the syndromic forms of ASD caused by rare mutations in single genes, epigenomic signatures have also provided some novel insights into the molecular pathogenesis into syndromes caused by copy number variants (CNVs), such as 15q11-q13 duplication [51, 52] or 16p11.2 deletion [53] syndromes, or aneuploidy, specifically Down syndrome (trisomy 21). These investigations, particularly those that have investigated brain and cell culture models, have provided important clues as to the genes within the large chromosomal regions that are responsible for the epigenetic changes observed (Fig. 1). While the specific mechanisms predicted to cause the epigenome-wide dysregulation observed in these different chromosomal syndromes are distinct, there are commonalities in the chromatin features, genes, and gene pathways impacted across disorders. Importantly, these patterns revealed from human genetics are beginning to complement the emerging evidence from animal models that neuronal maturation involves a cross-talk between de novo DNA methylation and histone modifications that mediate developmental plasticity through multiple mechanisms [54–56]. In zebrafish embryos, developmentally plastic “placeholder” nucleosomes, containing H2A.Z and H3K4me1, are anti-correlated with DNA methylation and their removal results in accumulation of DNA methylation [57]. In mammals, DNA hypomethylation is observed over developmentally poised “bivalent” chromatin containing histone H2A or H2A.Z that are both acetylated and ubiquitinated, and histone H3 that is methylated at both K4 and K27 residues [58, 59]. Specifically, recent studies have demonstrated that a *Dnmt3a* mutant that no longer binds to active chromatin results in progressive DNA hypermethylation across bivalent chromatin domains marked by H3K27me3 and de-repression of developmental regulatory genes in the adult hypothalamus [60]. An isoform-specific knockout of DNMT3A demonstrated that its N-terminal binding to ubiquitinated H2A (H2AK119ub) guides de novo DNA methylation over bivalent genes within the nervous system [61]. Furthermore, a *Dnmt3a* knockout specifically targeted to excitatory neurons resulted in stunted maturation of synapses, elevated H3K27me3 levels, and fetal-like DNA methylation patterns in the postnatal period [62]. Together, these experiments have shown the mechanistic connections between de novo DNA methylation and placeholder nucleosomes over poised chromatin that are being reflected in the broad epigenomic signatures of human neurodevelopmental disorders.

15q11-q13 duplication syndrome (Dup15q) is one of the most common CNVs identified in ASD cases and ASD comorbidity is observed in 85% of Dup15q cases [63]. Due to the same chromosomal breakpoints responsible for large deletions in 15q11-q13, Dup15q syndrome results from duplication that is either extrachromosomal or interstitial [64]. While duplications can occur on either parental chromosome, ASD is only observed in individuals with maternal duplication [63, 64]. The AS gene *UBE3A* is maternally expressed exclusively in neurons due to the paternal expression of the UBE3A antisense [63]. Because the 15q11-q13 locus is parentally imprinted, the strongest methylation signature observed in Dup15q brain is over a ~7 Mb region that is strikingly hypomethylated on the maternal allele by 20 kb window WGBS analysis, resulting in opposite DNA methylation directions in the 15q11-q13 deletion syndromes AS (hypermethylation) versus PWS (hypomethylation) and a more modest hypomethylation in Dup15q syndrome compared to controls [51]. The opposite pattern of maternal hypermethylation and paternal hypomethylation was observed specifically at CpG islands within the 7 Mb imprinted locus [51], reminiscent of what is observed on the inactive X chromosome in females [65]. The enrichment of both hypo- and hypermethylated probes over the imprinted 15q11-q13 locus in Dup15q syndrome was replicated in a 450k array analysis of three brain regions [52]. This 450k array study in brain also showed a significant overlap in the epigenomic signature between Dup15q and idiopathic ASD [52], a finding that was also replicated in a subsequent WGBS analysis of idiopathic ASD, Dup15q syndrome, and Rett syndrome [66].

Functional follow-up analyses using human neuronal cell line models revealed further insights into the potential mechanism responsible for the epigenomic signature of Dup15q syndrome and how multiple “hits” may impact the strength of the epigenomic signature. A human neuronal cell line model of Dup15q syndrome [67] was cultured in the presence or absence of the environmental pollutant polychlorinated biphenyl (PCB 95) [51], which had previously observed to be elevated in Dup15q brain samples compared to controls or idiopathic ASD [68]. Long-term clonal cultures of this Dup15q model acquired a second duplication on 22q12.3-q13.33, resulting in a multi-hit model of two chromosomal duplications plus the environmental exposure. Each additional hit increased the number of differentially methylated genes, mostly hypomethylated, with functions at the synaptic membrane [51]. Because most hypomethylated genes showed decreased expression in long-term cultures, an epigenetic change to chromatin modifications was investigated. Specifically, a known nuclear target of the ubiquitin ligase activity of UBE3A is RING1 [69], a component of the polycomb regulatory complex 1 (PRC1) repressor that is a ubiquitin ligase for the histone components H2A and H2A.Z [59]. Ubiquitinated H2A.Z is a poised developmental mark of large chromatin domains with lower levels of DNA methylation [59]. As shown in Fig. 1, bivalent H2A.Z marked the Dup15q hypomethylated genes and elevated levels of UBE3A correlated with reduced levels of RING1B. PCB 95 further reduced the levels of H2A.Z [51]. Together, these results suggested a multi-hit intersecting pathway between genetic susceptibility and an environmental exposure observed through the shared epigenomic signature.

Down syndrome (DS) is another neurodevelopmental disorder caused by a chromosomal copy number change, trisomy 21. Estimates of ASD incidence is DS have ranged from 5–39% dependent on the study [70]. Multiple studies have demonstrated that gene expression and epigenetic dysregulation in DS tissues occur genome-wide and are not necessarily enriched on chromosome 21 [71–76]. Gene loci hypermethylated in DS were most consistent across tissues, while hypomethylated loci were more tissue-specific. A WGBS study in newborn blood from DS versus other developmental delay or typically developing controls demonstrated a 28 kb domain on chromosome 21, spanning the *RUNX1* locus [73], which has been consistently hypermethylated across DS studies and tissues [71–76]. *RUNX1* encodes a developmental transcription factor important in both hematopoiesis and neurodevelopment [77, 78]. Interestingly, the regions hypomethylated in DS newborn blood were enriched for binding sites of RUNX1 [73]. Mechanistically, RUNX1 binding has been shown to demethylate its binding sites through recruitment of DNA demethylation enzymes (TET2, TET3, TDG, and GADD45) [79]. Therefore, these results are consistent with the mechanism of early life DS transcriptional and epigenetic dysregulation being in part due to both the overexpression and epigenetic modification of *RUNX1*.

Of the potential known epigenetic regulators encoded on chromosome 21, the DNA methyltransferase gene, *DNMT3L* has the most accumulated evidence supporting its role in the hypermethylated loci observed across tissue types in DS [80, 81]. DNMT3L is a catalytically inert homolog of DNMT3A and DNMT3B that serves as their protein partner and regulator of de novo DNA methylation [82, 83]. Overexpression of *DNMT3L* in in human neurons at three stages of differentiation demonstrated a hypermethylated signature shared with that of DS brain and other DS tissues [81]. In this study, *DNMT3L*-induced hypermethylation occurred predominantly at regions of bivalent chromatin that lose H3K4me3 during neuronal differentiation. In DNMT3L overexpressing cells, hypermethylation of *RUNX1* was observed in the neuroblast and differentiated neurons, but not those in process of differentiation [81]. Together, these findings (summarized in Fig. 1) demonstrate that early neuronal *DNMT3L* overexpression recreates a facet of the genome-wide DS DNA methylation signature, specifically the gene loci that consistently display hyper-methylation in DS such as *RUNX1*. These results also suggest that DNMT3L may be a major contributor to the hypermethylated bivalent chromatin signature in DS, while RUNX1 binding and demethylation may be a contributor to the blood-specific hypomethylated signature of DS. However, there are also consequences to cellular viability, DNA repair, and metabolism associated with aneuploidy independent of chromosome 21 dosage effects that have been demonstrated in fibroblasts from DS in common with other trisomies [84] that are also consistent with gene ontologies enriched in DS epigenomic signatures [72, 73, 81].

### Evidence for epigenetic signatures in human idiopathic ASD brain and surrogate tissues

While the discovery of distinct epigenomic signatures distinguishing syndromic forms of ASD is possible with a relatively low sample size and different tissue types besides brain, this same approach has been more challenging in finding a robust DNA methylation signature for idiopathic ASD. Postmortem brain samples from individuals with idiopathic ASD have been the tissue type used for initial EWAS, because of the predicted involvement in ASD symptomology. Several studies have used the Illumina Infinium 450k platform to investigate different brain regions (prefrontal and temporal cortex, cerebellum) from 6–12 ASD samples compared to matched controls, but no individual differentially methylated probe (DMP) reached genome-wide significance [52, 85–87]. However, using an approach of identifying differentially methylated regions (DMR), Ladd-Acosta et al identified three DMRs in temporal cortex (*PRRT1, ZNF57, C11orf21*) and one in cerebellum (*SDHAP3*). Furthermore, Wong et al. 2019 used a systems approach of grouping comethylated gene loci into modules, identifying a number of significant gene ontologies associated with idiopathic ASD, including homophylic adhesion, synapse part, and calcium ion binding [52] that were convergent with other idiopathic ASD and Dup15q syndrome analyses [51, 85, 86].

The use of a sequencing-based WGBS approach allowed the discovery of 483 DMRs in idiopathic ASD prefrontal cortex, although this number of regions was 5–10x lower that those identified in two syndromic forms of ASD, Dup15q and Rett syndromes by the same analyses [66]. In addition to enrichment for genes with functions in nervous system development, ASD DMRs were also enriched for regions of open chromatin in microglia, the major immune cell type in brain [66]. In this study, ASD DMRs were also enriched for binding sites of known methyl-sensitive transcription factors, including IRF3, NRF1, YY1, and RFX5. Furthermore, a group of 65 genes overlapped for enrichment in DMRs identified in all three disorders (idiopathic ASD, Dup15q, Rett) that included many of those with known genetic risk for ASD (*OTX1*, *GRIK4*, and *ADCY5*), as well as also differentially expressed in ASD (*MYT1L*, *ZFHX3*, *RBFOX1*) [66]. Furthermore, the genes associated with ASD DMRs in this study showed significant overlaps with those identified from other transcriptome and epigenomic studies in ASD cerebral cortex [66]. Together, these studies support an epigenomic signature associated with idiopathic ASD in the prefrontal cortex that shares some similarities to those of syndromic ASD.

While these biological insights gained from brain EWAS studies have been important, there are many limitations to these studies, including the limited number of samples and the inability to control for confounding variables, such as medications and time and cause of death. Furthermore, the inaccessibility of sampling brain DNA from living individuals makes it not useful as a predictive biomarker. Therefore, multiple studies have attempted to use EWAS in accessible tissues such as blood, with limited success. Hannon et al performed a 450k EWAS on DNA samples isolated from whole blood matched to 4 brain regions (prefrontal cortex, entorhinal cortex, superior temporal gyrus, and cerebellum) from 122 typically developing individuals, concluding that interindividual variation in whole blood was not a strong predictor of interindividual variation in the brain [88]. This finding is consistent with two relatively large studies that did not identify a distinguishable epigenomic signature of idiopathic ASD from blood samples obtained in children after diagnosis [89, 90]. Though these studies failed to identify any individual DMPs at genome-wide significance, there were correlations observed between top DMPs and quantitative autistic trait scores in one study [90] or with some brain DMPs in another [89]. A 450k EWAS study of 95 buccal epithelial samples from ASD versus TD individuals born to mothers ≥35 years identified 9 genes and synaptic pathways associated with ASD from alternative methods of DMRs and comethylated modules [91]. A large 450k EWAS using newborn blood spots was successful in associating polygenic risk score for ASD with methylation variation at some specific chromosomal loci, but did not identify any DMP associated with ASD alone [92].

**Fig. 2:**
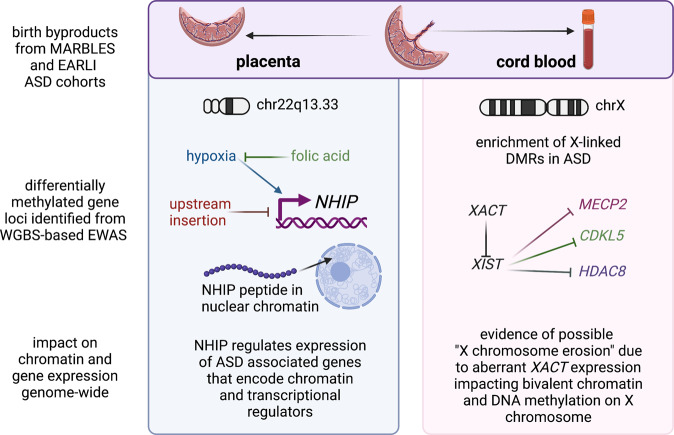
Mechanistic insights from epigenomic signatures of idiopathic ASD newborn surrogate tissues. At birth, the fetal derived byproducts of placenta and cord blood are usually discarded, but were collected as part of the prospective MARBLES and EARLI cohorts of enriched idiopathic ASD risk. Since they are derived from different lineages of the early embryo, placenta and cord blood from the same individuals have each revealed distinct mechanisms from their epigenomic signatures. Analyses of placenta (left panel) resulted in the discovery of the neuronal hypoxia inducible, placenta associated (*NHIP*) locus on 22q13.33. *NHIP* methylation levels were associated with both genetics (upstream insertion) and folic acid (prenatal vitamin use in first pregnancy month). In differentiated human neurons, hypoxia and resulting oxidative stress increase *NHIP* levels. ASD placenta and brain samples show significantly lower *NHIP* levels, suggesting a protective effect. *NHIP* is primate-specific and encodes a conserved micropeptide that associates with nuclear chromatin. *NHIP* elevated transcript levels alter many downstream gene targets enriched for regulators of chromatin and ASD genetic risk. In contrast, the idiopathic ASD epigenomic signature from cord blood samples revealed an enrichment for X-linked differentially methylated genes, as well as those involved in early neurodevelopment. Differential methylation was observed at the *XACT* locus, a primate-specific noncoding RNA expressed exclusively from the active X chromosome that represses *XIST* during the establishment of X chromosome inactivation. In human pluripotent stem cell culture, *XACT* is implicated in the phenomenon of X chromosome erosion, characterized by the partial loss of epigenetic silencing of X-linked genes and regions of bivalent chromatin on the X chromosome in females. While many X linked genes implicated in genetic risk for ASD were observed to be differentially methylated in ASD cord blood DNA, three specific examples (*MECP2*, *CDKL5*, and *HDAC8*) involved in syndromic forms of ASD are shown. Created with BioRender.com.

Potentially more promising for identifying predictive epigenetic biomarkers for ASD are those studies that have performed EWAS on newborn samples obtained at delivery in prospective studies of enriched ASD risk. Two prospective cohorts in the US have recruited mothers of a prior child with ASD who are pregnant, thus enriching ASD diagnosis ten-fold compared to population risk: Markers of Autism Risk in Babies – Learning Early Signs (MARBLES) and Early Autism Risk Longitudinal Investigation (EARLI). Both studies collect and store maternal biospecimens during pregnancy, cord blood and placenta at delivery, and follow up children through 36 months with biospecimen collections and quantitative evaluations of social and cognitive outcomes [93, 94]. Both array-based and EWAS studies have been used to investigated ASD epigenomic signatures of idiopathic ASD using samples from these cohorts [95–103]. Because the use of WGBS allowed the more extensive coverage of regions of the genome that are the most variable between individuals (discussed above), the focus of this summary is on the two most recent and largest studies using WGBS-based EWAS on placenta [96] versus cord blood^39^ that have led to new mechanistic insights into ASD (Fig. 2).

Using MARBLES as the discovery cohort and EARLI as the replication cohort, Zhu et al 2022 analyzed a total of 204 placenta samples (83 ASD, 107 controls) using WGBS to identify ASD-associated methylation changes [96]. 134 ASD associated DMRs were identified in the discovery group that were enriched for bivalent chromatin regions in placenta and enhancers in fetal brain. Remarkably, a large block of CpGs in 22q13.33 was significantly hypomethylated in ASD and this finding replicated across both MARBLES and EARLI cohorts. This 22q13.33 block of comethylated regions was previously identified as a CoRSIV and a region of bivalent chromatin in placenta, but did not contain any well characterized genes. There was a transcript within the 22q13.33 block annotated as *LOC105373085* which additional functional studies showed to be expressed in brain and induced in response to neuronal differentiation and oxidative stress. This transcript was therefore renamed *NHIP* for neuronal hypoxia inducible, placenta associated. Using RNAseq on both postmortem ASD and control brain samples as well as *NHIP*-overexpressing cell lines, *NHIP* was shown to regulate expression of other known ASD-risk genes involved in chromatin and transcriptional regulation. A common structural variant within the *NHIP* locus was independently associated with increased ASD risk, reduced expression of *NHIP*, and reduced methylation. Interestingly, placentas from mothers who took a folic acid vitamin in the first month of pregnancy, a known protective anti-oxidant methyl-donor [104, 105], showed increased *NHIP* methylation, essentially counteracting the genetic risk at *NHIP* [96]. Functionally, *NHIP* encodes a 20 amino acid peptide that localizes to neuronal nuclei in human brain. While this peptide is well conserved amongst primates, *NHIP* is not detected in other mammals, suggesting a recent evolved function in the response to hypoxia and oxidative stress in primates [96]. Within primates, humans have evolved the highest brain-to-body ratio, with a human fetal brain consuming up to 60% of the body’s oxygen and energy consumption, despite making up ~13% of body mass [106]. Together, these results suggest that transient NHIP expression in response to hypoxia is neuroprotective.

In contrast to placenta which is derived from the trophectoderm layer of the preimplantation embryo in all mammals, cord blood is derived from the hematopoietic cell lineage of the embryo proper [107]. So perhaps not surprisingly, distinct properties of ASD associated DMRs were identified in the analysis of cord blood compared to placenta using similar WGBS and DMR approaches [95, 96, 98]. Unlike the placental analysis for which the variable of offspring sex was adjusted for [96], cord blood WGBS analysis was most informative when samples were stratified by sex because of the strong enrichment for cord blood ASD DMRs on the X chromosome [95]. Subjects in this study were 74% male from both MARBLES and EARLI high-familial risk prospective cohorts. Replication across different cohorts identified 537 ASD DMR genes in males and 1762 ASD DMR genes in females by gene association. ASD DMR genes identified from cord blood were significantly enriched for brain and embryonic expression and identification in prior epigenetic studies of ASD in post-mortem brain. Like what ASD DMRs revealed in postmortem brain, those identified in cord blood were significantly enriched for binding sites of methyl-sensitive transcription factors relevant to fetal brain development. The major finding that ASD DMRs identified brain and early developmental functions rather than immune functions [95] was in distinct contrast to a transcriptome analysis of the same samples which only identified blood and immune functions [108]. Furthermore, autosomal ASD DMRs from cord blood were significantly enriched for promoter and bivalent chromatin states in both sexes, while sex differences were observed for X-linked ASD DMRs [95]. Interestingly, the enrichment of differentially methylated genes on the X chromosome included the primate-specific noncoding transcript *XACT* [95], which is expressed from the active X chromosome in males and females and has been implicated in the phenomenon of X chromosome erosion in human pluripotent cells [109, 110]. Furthermore, many of the genes associated with ASD DMRs in cord blood were known syndromic ASD risk genes [95], including *MECP2* and *CDKL5* (Rett syndrome) [111], as well as histone deacetylase *HDAC8* (Cornelia de Lange syndrome 5) [112].

### Future perspectives

The multiple lines of evidence presented above have established that epigenetic changes in the form of distinct DNA methylation signatures characterize both syndromic and idiopathic neurodevelopmental disorders on the autism spectrum. There is also strong evidence that in general, most inter-individual variation in DNA methylation is best explained by GxE models, meaning that common genetic risk is predicted to dynamically interact with common but variable environmental factors. Since most ASD cases are unlikely to have a single genetic or environmental cause, the use of epigenomic signatures to detect the multivariate intersection is likely to improve understanding of common genetic risk for ASD. Importantly, the DNA methylome signature of ASD polygenic risk is highly supportive of most common risk involving GxE more than other neuropsychiatric disorders [40]. This is insightful considering a recent study showing that polygenic risk score for schizophrenia is only predictive for those with early life complications [112]. Thus, being able to accurately interpret the unique signatures of the DNA methylome at birth within the heterogeneous mixture of ASD offers important insights to improved diagnosis and therapy.

Since ASD is currently diagnosed by behavioral testing that does not detect all cases until 3 years of age [113], there is a current need for early biomarkers of ASD risk, so that at risk toddlers may receive behavioral interventions at an age where they are most effective [114, 115]. In the future, newborn genetic screens may be widened to include the known syndromic forms of ASD [116, 117]. However, since these genetic tests need to be performed one at a time on limited DNA isolated from newborn blood spots, a more efficient screen could be an epigenetic one, perhaps using an algorithmic predictive strategy like EpiSign [117], discussed above. For such a screening to be both sensitive and specific for both syndromic as well as idiopathic forms of ASD, replication with larger sample sizes and careful design of the most appropriate genomic regions to assay is imperative to glean the benefits of both array-based and sequencing-based platforms (Box 2). The latest genomic advancements, including the entire telomere to telomere (T2T) human genome [118, 119] and DNA methylome [120] maps should be used when choosing the regions with the most inter-individual variation and differences between ASD and control from the analyses of early life tissues. Cell-specific methylome maps in brain [121–123] are another emerging resource also of critical importance for designing improved assays for clinical use. Single cell technologies of brain cell transcriptomes and epigenomes have been reviewed elsewhere [123, 124]. More research is clearly needed in prospective human cohorts in which samples and data collected during pregnancy and childbirth are stored and widely distributed. This is a major goal of the Environmental effects of Child Health Outcomes (ECHO) US-wide birth cohort study [125] that has the potential to be used for future EWAS and transgenerational investigations [126] into intersections of genetic and environmental risk factors for ASD and other adverse health outcomes.

Advances in machine learning are already beginning to be used to determine the most sensitive and specific predictors of ASD diagnosis from large-scale EWAS datasets [127–129]. The major challenge to the field at this stage is that the training on small sample sizes become problematic in overfitting data based on too little and likely highly biased data inputs. Major efforts towards recruitment of larger numbers of research subjects from diverse and medically underserved populations is therefore important if the resulting predicted are going to be beneficial for screening of all newborns. Ideally, multiple layers of information about each subject, including genetic variants, DNA methylation signatures, measured metabolite levels, brain imaging measurements, as well as a variety of surveyed information concerning social determinants of health would be integrated into machine learning predictive algorithms. In addition to simple yes/no diagnoses, these could potentially become sophisticated enough to predict some of the clinical variability within syndromic disorders, such as epilepsy within Dup15q, or comorbidities within idiopathies ASD.

Moving to an even earlier stage of potential preventative intervention that the newborn stage, prenatal screening from cell free fetal DNA within maternal blood holds potential for early identification and interventions to improve brain development during pregnancy for at risk fetuses. Currently, non-invasive prenatal screening is performed in the first trimester as an alternative to chorionic villus sampling for prenatal screening of Down syndrome. This procedure involves sampling and sequencing the cell-free fetal DNA (cffDNA) fraction from blood of pregnant mothers [130, 131]. cffDNA originates from the trophoblasts of the placenta, based on genetic evidence from cases of anembryonic pregnancies or confined placental mosaicism [132–134]. DNA methylation evidence also has demonstrated that cffDNA contains partially methylated domains [135, 136] which are uniquely characteristic of placenta [137, 138]. Currently, cancer epigenomic signatures from cell free DNA derived from tumor cells is showing promise for early detection and tissue origin of multiple cancer types [139–141]. Therefore, profiling epigenomic signatures or specific placental gene loci such as *NHIP* from cffDNA within maternal blood could provide an early marker of ASD risk in the child. Early identification of risk could prompt existing interventions during pregnancy that reduce medical complications and environmental exposures. Some preventative epigenomic screenings could even be performed pre-conception, as there has be some predictive success within small studies of sperm methyome differences in fathers of ASD offspring [103, 142]. There are clearly ethical concerns and limitations to pre-conception and prenatal epigenomic screening for ASD risk to avoid the negative impacts associated with false positives. Clearly, more research is needed on epigenomic screening and preventative intervention of ASD risk.

Following up on specific mechanistic insights provided by epigenomic signatures from both syndromic and idiopathic ASD early life samples is expected to inform future therapies and early interventions. Future investigations should seek to understand the overlap between ASD-associated DNA methylation signatures with additional epigenetic layers, including histone acetylation and long noncoding RNAs identified as differential in ASD brain [143, 144]. Comparisons to brain organoid cultures derived from ASD patient-derived or genetically engineered stem cells (reviewed in [145, 146]) are also expected to inform mechanism of developmental epigenetic changes in ASD. Since the epigenomic signatures provided from bulk tissues are often complex, use of emerging single cell approaches to define ASD signatures in perinatal tissues such as placenta and cord blood is expected to be informative. For instance, single cell methylomes have been instrumental in dissecting our specific neuronal cell types in human brain [147, 148]. These approaches have recently enabled multi-omic analyses of the methylome, transcriptome, chromatin accessibility, and chromatin loops for human cortical cell types [121]. When these multi-omic cellular maps were compared to GWAS loci for neuropsychiatric traits, they demonstrated a significant enrichment for closed chromatin within layer 6 excitatory neurons, for example. The future use of multi-omic single cell methods in comparisons of ASD to control samples is expected to shed additional light on convergent mechanisms in the pathogenesis of ASD, including the dynamic changes to DNA methylation and bivalent chromatin highlighted in this review.

But in addition to the cell type specific mechanisms, there may also be some tissue independent and universal defining mechanisms across different etiologies of ASD that are revealed from epigenomic signatures of syndromic and/or idiopathic ASD. An example discussed in this review is the *NHIP* regulatory gene locus that was discovered in placenta, but highly expressed in brain and responsive to hypoxia in differentiated human neurons [96]. Hypoxia and oxidative stress are pathways in common to multiple environmental insults as well as placental complications during pregnancy. Since elevated *NHIP* expression in response to oxidative stress appears to be protective for the development of ASD, the *NHIP* gene or encoded micropeptide could be promising as a therapeutic approach during pregnancy or early infancy in high-risk populations. In summary, the discoveries made from epigenomic investigations using the latest genomic approaches are likely to yield clinically relevant advancements in the future that would not have been possible through traditional genetic approaches such as GWAS.
